# Diverse Host-Seeking Behaviors of Skin-Penetrating Nematodes

**DOI:** 10.1371/journal.ppat.1004305

**Published:** 2014-08-14

**Authors:** Michelle L. Castelletto, Spencer S. Gang, Ryo P. Okubo, Anastassia A. Tselikova, Thomas J. Nolan, Edward G. Platzer, James B. Lok, Elissa A. Hallem

**Affiliations:** 1 Department of Microbiology, Immunology, and Molecular Genetics, University of California, Los Angeles, Los Angeles, California, United States of America; 2 Department of Pathobiology, University of Pennsylvania, Philadelphia, Pennsylvania, United States of America; 3 Department of Nematology, University of California, Riverside, Riverside, California, United States of America; University of Glasgow, United Kingdom

## Abstract

Skin-penetrating parasitic nematodes infect approximately one billion people worldwide and are responsible for some of the most common neglected tropical diseases. The infective larvae of skin-penetrating nematodes are thought to search for hosts using sensory cues, yet their host-seeking behavior is poorly understood. We conducted an in-depth analysis of host seeking in the skin-penetrating human parasite *Strongyloides stercoralis*, and compared its behavior to that of other parasitic nematodes. We found that *Str. stercoralis* is highly mobile relative to other parasitic nematodes and uses a cruising strategy for finding hosts. *Str. stercoralis* shows robust attraction to a diverse array of human skin and sweat odorants, most of which are known mosquito attractants. Olfactory preferences of *Str. stercoralis* vary across life stages, suggesting a mechanism by which host seeking is limited to infective larvae. A comparison of odor-driven behavior in *Str. stercoralis* and six other nematode species revealed that parasite olfactory preferences reflect host specificity rather than phylogeny, suggesting an important role for olfaction in host selection. Our results may enable the development of new strategies for combating harmful nematode infections.

## Author Summary

Parasitic worms are a significant public health problem. Skin-penetrating worms such as hookworms and the human threadworm *Strongyloides stercoralis* dwell in the soil before infecting their host. However, how they locate and identify appropriate hosts is not understood. Here we investigated the host-seeking behavior of *Str. stercoralis*. We found that *Str. stercoralis* moves quickly and actively searches for hosts to infect. We also found that *Str. stercoralis* is attracted to human skin and sweat odorants, including many that also attract mosquitoes. We then compared olfactory behavior across parasitic worm species and found that parasites with similar hosts respond similarly to odorants even when they are not closely related, suggesting parasitic worms use olfactory cues to select hosts. A better understanding of host seeking in skin-penetrating worms may lead to novel control strategies.

## Introduction

**Figure 1:**
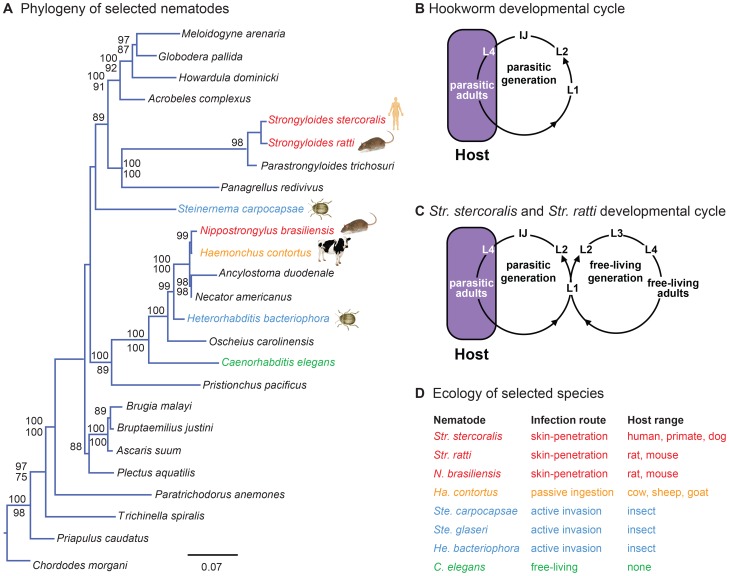
Phylogenetic relationships and life cycles of parasitic nematodes. **A**. Phylogeny of selected nematode species. Phylogenetic analysis is from Dillman *et al.*, 2012 [22]. Species used in the present study are highlighted. Red = skin-penetrating mammalian-parasitic nematode; gold = passively ingested mammalian-parasitic nematode; blue = entomopathogenic nematode; green = free-living nematode. For each of the selected species, icons depict one of their common hosts (human, rat, beetle, or cow). Phylogenetic relationships are based on ML and Bayesian analyses of nearly complete SSU sequences. Values above each branch represent Bayesian posterior probabilities; ML bootstrap indices appear below each branch. Values lower than 75 are not reported. *Priapulus caudatus* and *Chordodes morgani* were defined as outgroups. Detailed methods for phylogenetic tree construction are provided in Dillman *et al.*, 2012 [22]. **B–C**. Life cycles of skin-penetrating nematodes. **B**. Hookworms must infect a new host every generation. IJs infect hosts by skin-penetration. Nematodes develop to adulthood, reproduce, and lay eggs inside the host. Eggs are excreted in host feces and develop into IJs, which find and infect new hosts. **C**. *Str. stercoralis* and *Str. ratti* can develop through a single generation outside the host. Some larvae excreted in host feces develop into IJs; others develop into free-living adults that mate and reproduce outside the host. All progeny of free-living adults develop into IJs, which find and infect new hosts. L1–L4 are larval stages; IJ = infective juvenile. **D**. Ecology of selected nematode species.

Skin-penetrating nematodes such as the threadworm *Str. stercoralis* and the hookworms *Ancylostoma duodenale* and *Necator americanus* (Figure 1A) are intestinal parasites that infect approximately 1 billion people worldwide. Infection with skin-penetrating worms can cause chronic gastrointestinal distress as well as stunted growth and long-term cognitive impairment in children. Moreover, *Str. stercoralis* infection can be fatal for immunocompromised individuals and infants [1]. *Str. stercoralis* is endemic in tropical and sub-tropical regions throughout the world, including the United States, and is estimated to infect 30–100 million people worldwide [2]. Infection rates in rural and semi-rural areas are often high, particularly among children. For example, a recent study found that 25% of school children in semi-rural Cambodia were infected with *Str. stercoralis* [3]. A better understanding of how skin-penetrating worms target human hosts could lead to new strategies for preventing infections.

Skin-penetrating nematodes are infective only during a particular stage of their life cycle called the infective juvenile (IJ), a developmentally arrested third larval stage analogous to the *C. elegans* dauer [4]. IJs inhabit the soil and infect by skin penetration, often through the skin between the toes. Inside the host, IJs migrate through the circulatory system to the lungs, are coughed up and swallowed, and develop to adulthood in the intestine [1]. IJs may also reach the intestine using other migratory routes [5]. Adult nematodes reproduce in the intestine, and eggs or young larvae are excreted in feces. In the case of hookworms, young larvae develop into IJs, which find and infect new hosts (Figure 1B). In the case of *Strongyloides* species, some larvae develop into IJs and others develop into free-living adults. In the human parasite *Str. stercoralis* and the rat parasite *Str. ratti*, which are subjects of this study, all progeny of free-living adults develop into IJs (Figure 1C). Some species of *Strongyloides*, such as the dog and cat parasite *Str. planiceps*, can undergo a limited number of sequential free-living generations [6]. Thus, *Strongyloides* can develop through at least one free-living generation outside the host. *Str. stercoralis* can also cycle through multiple parasitic generations in the same host, resulting in a potentially fatal disseminated infection [1].

Little is known about the process by which skin-penetrating nematodes find hosts [7]. IJs of some skin-penetrating species respond to heat and sodium chloride [8]–[12], suggesting a role for thermosensation and gustation in host seeking. In addition, *Str. stercoralis* is attracted to human blood serum and sweat [10], [12], while *Str. ratti* is attracted to mammalian blood serum [13]. It has long been speculated that olfaction may be important for host seeking, since animals emit unique odor blends that could confer species-specificity [7]. However, the only specific odorant that has so far been found to elicit a response from a skin-penetrating nematode is urocanic acid, a component of mammalian skin that attracts *Str. stercoralis* [14]. Thus, the extent to which skin-penetrating nematodes use olfactory cues to locate hosts is unclear.

Here we examined the host-seeking strategies and sensory behaviors of the human parasite *Str. stercoralis* as well as two other species of skin-penetrating nematodes, the rat parasites *Str. ratti* and *Nippostrongylus brasiliensis* (Figure 1A, D). We compared their behaviors to those of five other nematode species with diverse lifestyles and ecological niches: the passively ingested ruminant-parasitic nematode *Haemonchus contortus*; the entomopathogenic nematodes (EPNs) *Heterorhabditis bacteriophora, Steinernema glaseri*, and *Steinernema carpocapsae*; and the free-living nematode *Caenorhabditis elegans* (Figures 1A, D). This across-species analysis was used to fit the behaviors of skin-penetrating nematodes into an ecological framework, and to identify species-specific behavioral differences that reflect differences in phylogeny, host range, or infection route. We found that different species of mammalian-parasitic nematodes employ diverse host-seeking strategies, with the human parasite *Str. stercoralis* being a cruiser that actively seeks out hosts. We found that *Str. stercoralis* and the other skin-penetrating nematodes are attracted to skin and sweat odorants, while the passively ingested ruminant parasite *Ha. contortus* is attracted to the smell of grass. By comparing odor response profiles across species, we found that olfactory preferences reflect host specificity rather than phylogeny, suggesting a critical role for olfaction in the process of host finding and appropriate host selection. Our results provide insight into how skin-penetrating nematodes locate hosts to infect.

## Results/Discussion

### Mammalian-parasitic nematodes vary in their movement patterns

**Figure 2:**
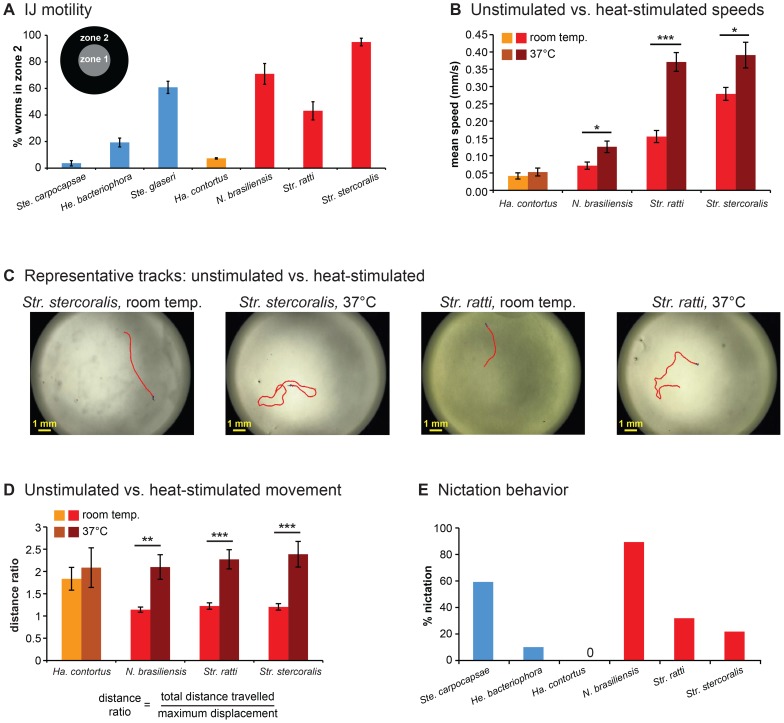
Foraging behaviors of skin-penetrating nematodes. **A**. IJ motility in the absence of chemosensory stimulation. Motility varies across species (*P*<0.0001, one-way ANOVA), with *Str. stercoralis* being the most active (*P*<0.01, one-way ANOVA with Tukey-Kramer post-test). n = 6–9 trials for each species. For this graph and subsequent graphs with multiple species, red = skin-penetrating; gold = passively ingested; blue = entomopathogenic. Of the three entomopathogenic species, *Ste. carpocapsae* is considered an ambusher, *Ste. glaseri* is considered an active cruiser, and *He. bacteriophora* is considered a less active cruiser [15]. Statistical analysis is shown in Table S1. **B**. Unstimulated vs. heat-stimulated mean speeds of mammalian-parasitic IJs. Heat-stimulated IJs were exposed to an acute 37°C stimulus and tracked at 37°C. ***, *P*<0.001; *, *P*<0.01, unpaired t test or Mann-Whitney test. n = 5–10 trials for each species. **C–D**. Heat stimulates local search behavior. **C**. Representative tracks for *Str. stercoralis* and *Str. ratti* from 20 s recordings at room temperature versus 37 s recordings at room temperature versus 37°C. **D**. Movement patterns at room temperature versus 37°C. Distance ratios were calculated as the total track length divided by the maximum displacement attained during the 20 s recording period. A distance ratio of 1 indicates travel in a straight line s recording period. A distance ratio of 1 indicates travel in a straight line; a distance ratio of >1 indicates a curved trajectory. ***, *P*<0.001; **, *P*<0.01, Mann-Whitney test. n = 5–10 trials. **E**. Nictation frequencies of IJs. Nictation was defined as standing or waving behavior of at least 5 s in duration over the course of a 2 min period. Nictation frequencies varied among species (*P*<0.0001, chi-square test). *N. brasiliensis* showed a nictation frequency comparable to *Ste. carpocapsae* (*P*>0.05, chi-square test with Bonferroni correction) and greater than *Str. stercoralis* or *Str. ratti* (*P*<0.01, chi-square test with Bonferroni correction). Statistical analysis is shown in Table S4. n = 20–28 IJs for each species. For all graphs, error bars indicate SEM.

To gain insight into the host-seeking strategies used by mammalian-parasitic nematodes, we first examined their movement patterns in the absence of chemosensory stimuli. We compared their movement patterns to those of EPNs, which use well-characterized host-seeking strategies: some are “cruisers” that actively search for hosts, some are “ambushers” that wait for passing hosts, and some use an intermediate strategy [9], [15]. We first examined motility using an assay in which IJs were allowed to distribute on an agar plate in the absence of chemosensory stimuli for one hour and the location of IJs on the plate was recorded. We found that the motility of skin-penetrating IJs resembled that of EPN cruisers, with the human parasite *Str. stercoralis* being the most active (Figure 2A). By contrast, the motility of *Ha. contortus* resembled that of the ambushing EPN *Ste. carpocapsae* (Figure 2A). Thus, skin-penetrating IJs appear to be more active than passively ingested IJs.

To investigate the host-seeking strategies of skin-penetrating nematodes in more detail, we examined unstimulated movement of IJs using automated worm tracking [16]. We found that parasitic IJs vary dramatically in their crawling speeds, with the human parasite *Str. stercoralis* moving more rapidly than the other species tested (Figure S1A). The mean speeds of the skin-penetrating rat parasites were comparable to that of the most active EPN, *Ste. glaseri*, while the mean speed of *Ha. contortus* resembled that of the less active EPNs (Figure S1A). Turn probability also varied among species but did not correlate with speed (Figure S1B). Some but not all species crawled significantly faster following mechanical stimulation, and in fact the maximum speeds attained by *Str. stercoralis*, *Str. ratti*, and *Ste. glaseri* following mechanical stimulation were similar (Figure S1C–D, Movies S1 and S2). Thus, at least some of the differences in basal crawling speeds among species reflect differences in movement strategy rather than differences in the inherent speeds at which the IJs are capable of crawling.

The fact that *Str. stercoralis* has a higher basal speed than *Str. ratti* and *N. brasiliensis* is consistent with the possibility that host-seeking strategy evolved independently in these species to accommodate host behavior and ecology. *Str. ratti* and *N. brasiliensis* are parasites of nesting rodents, which are highly focal with circumscribed resting places. Since parasite transmission likely occurs within the confines of the nest, rapid mobility may not provide an adaptive advantage for these parasites. By contrast, *Str. stercoralis* is a parasite of humans, primates, and dogs, all of which are highly mobile. Rapid mobility may be necessary for *Str. stercoralis* to accommodate the mobility of its hosts.

### Heat increases crawling speed and stimulates local searching in skin-penetrating nematodes

Heat is emitted by all mammals and is a known sensory cue for some mammalian-parasitic nematodes, including *Str. stercoralis* [11]. We therefore examined the responses of the mammalian-parasitic IJs to a 37°C heat stimulus. We found that the skin-penetrating nematodes increased their crawling speed in response to thermal stimulation, while the passively ingested nematode *Ha. contortus* did not (Figure 2B). Skin-penetrating nematodes may increase their speed in response to heat to maximize the likelihood of encountering host skin.

A comparison of IJ movement patterns at room temperature versus 37°C revealed that skin-penetrating IJs show dramatically different movement patterns at the different temperatures. The trajectories of individual IJs were relatively straight at room temperature but highly curved at 37°C (Figure 2C). To quantify these differences, we calculated a distance ratio consisting of the total distance travelled divided by the maximum displacement achieved. We found that all three species of skin-penetrating nematodes showed greater distance ratios at 37°C compared to room temperature (Figure 2D). These results suggest that heat may act as a cue that signifies host proximity and stimulates local searching. However, we note that the temperature at the surface of human skin is 32–35°C [17], and IJ movement within this temperature range remains to be examined.

### Nictation behavior varies among mammalian-parasitic nematodes

An important component of host-seeking strategy for many parasitic nematodes is nictation, a behavior in which the worm stands on its tail and waves its head to facilitate attachment to passing hosts [9]. We examined the nictation behavior of mammalian-parasitic nematodes by performing nictation assays on an “artificial dirt” substrate consisting of dense agar with near-microscopic pillars [18], since IJs are not capable of standing on standard agar plates due to the high surface tension on the plates [18]. We found that nictation frequencies varied among species. *N. brasiliensis* showed a high nictation frequency comparable to that of the ambushing EPN *Ste. carpocapsae* (Figure 2E and Movie S3), suggesting that it spends most of its foraging time nictating. By contrast, the *Strongyloides* species showed much lower rates of nictation (Figure 2E and Movie S4), suggesting they spend most of their foraging time crawling. *Ha. contortus* did not nictate on the artificial dirt substrate or any other substrate tested (see Materials and Methods), suggesting it may not be capable of nictating.

### Mammalian-parasitic nematodes utilize diverse host-seeking strategies

Taken together, our results suggest that mammalian-parasitic nematodes employ diverse host-seeking strategies. The skin-penetrating *Strongyloides* species appear to be cruisers that are highly mobile and tend to crawl rather than nictate. By contrast, the passively ingested nematode *Ha. contortus* appears to be an ambusher that displays little unstimulated movement. *N. brasiliensis* can exhibit rapid, prolonged movement comparable to that of the cruisers but tends to nictate rather than crawl, suggesting it is also an ambusher. However, we note that foraging strategy is in some cases substrate-dependent, and different strains of a species can exhibit different host-seeking behaviors [19], [20]. Thus, we cannot exclude the possibility that the host-seeking strategies of these species may vary under conditions not tested here.

### *Str. stercoralis* is attracted to human-emitted odorants

**Figure 3:**
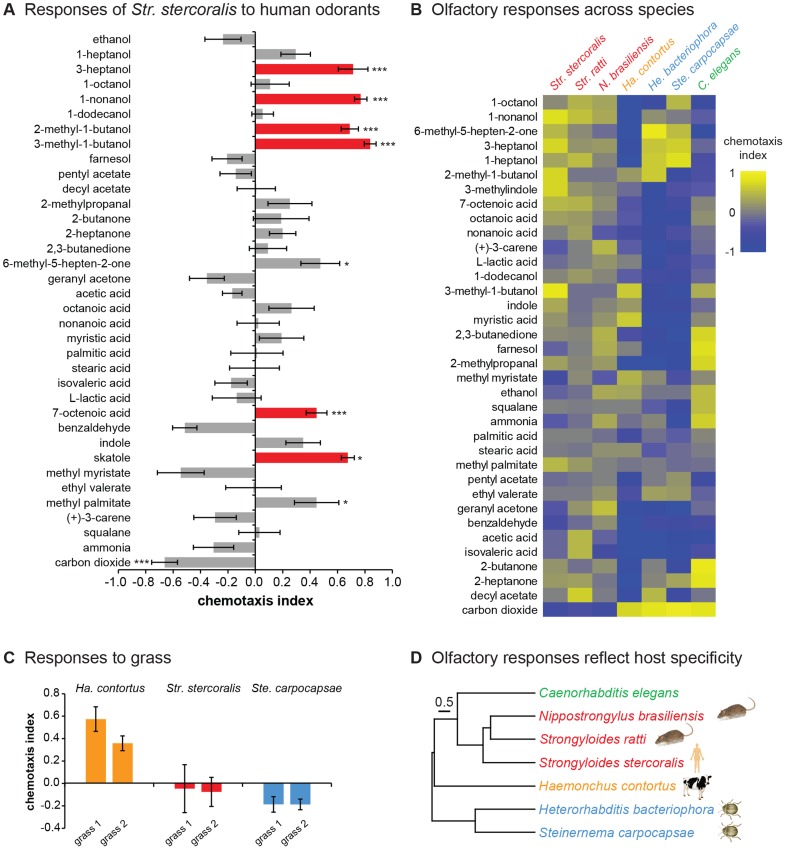
Olfactory responses of mammalian-parasitic nematodes. **A**. *Str. stercoralis* is attracted to a number of human-emitted odorants. Red = attractants for *Str. stercoralis* that also attract anthropophilic mosquitoes [31], [52]–[58]. n = 6–23 trials per odorant. *Str. stercoralis* did not respond to the chemotaxis controls (Figure S3). *, *P*<0.05; ***, *P*<0.001 relative to control, t-test (CO_2_ vs. air and L-lactic acid vs. H_2_O) or one-way ANOVA with Bonferroni post-test (all other odorants vs. paraffin oil). **B**. Olfactory responses across species. Response magnitudes are color-coded according to the scale shown to the right of the heat map, and odorants are ordered based on hierarchical cluster analysis. n = 6–14 trials for each odorant-species combination. Each species exhibited a unique odor response profile (*P*<0.0001, two-way ANOVA with Tukey's post-test). Data for responses of EPNs and *C. elegans* to 10% CO_2_ are from Dillman *et al.*, 2012 [22]. Red = skin-penetrating; gold = passively ingested; blue = insect-parasitic; green = free-living. **C**. Responses of *Ha. contortus* to grass odor. Responses to the odors of two different grass samples were examined. n = 8–17 trials for each sample. **D**. Olfactory preferences reflect host specificity rather than phylogeny. The behavioral dendrogram was constructed based on the odor response profiles of each species. Hierarchical cluster analysis was performed using UPGMA (Unweighted Pair Group Method with Arithmetic Mean). Euclidean distance was used as a similarity measure. Hosts (humans, ruminants, rodents, or insects) for each species are indicated. Coph. Corr. = 0.96. For all graphs, error bars indicate SEM.

EPNs have been shown to use a diverse array of insect volatiles and herbivore-induced plant volatiles for host finding [21]–[30]. By contrast, only one odorant has so far been identified as an attractant for *Str. stercoralis* [14]. We therefore tested the extent to which *Str. stercoralis* displays directed movement in response to human-emitted volatiles. We examined the responses of *Str. stercoralis* IJs to a large panel of odorants, most of which are known to be emitted by human skin, sweat, and skin microbiota (Table S5). Responses were examined using a chemotaxis assay (Figures S2 and S3) [21], [22]. We found that *Str. stercoralis* was strongly attracted to a number of these odorants (Figure 3A). Nearly all of the attractants we identified for *Str. stercoralis* also attract anthropophilic mosquitoes (Figure 3A), suggesting that nematodes and mosquitoes target humans using many of the same olfactory cues. While many of the human-emitted odorants that attracted *Str. stercoralis* are also emitted by other mammals, 7-octenoic acid is thought to be human-specific [31] and may be used by *Str. stercoralis* to target humans. *Str. stercoralis* and disease-causing mosquitoes are co-endemic throughout the world [2], and our results raise the possibility of designing traps that are effective against both parasites.

We also examined responses to carbon dioxide (CO_2_), which is emitted by aerobic organisms in exhaled breath and is an attractant for many parasites, including EPNs [9], [21], [22]. We found that *Str. stercoralis* was repelled by CO_2_ at high concentrations and neutral to CO_2_ at low concentrations, suggesting that CO_2_ is not a host attractant (Figure 3A and Figure S4A). These results are consistent with the fact that *Str. stercoralis* infects by skin penetration, and only low levels of CO_2_ are emitted from skin [32]. However, some EPNs respond synergistically to mixtures of CO_2_ and other odorants [33], and we cannot exclude the possibility that *Str. stercoralis* is attracted to CO_2_ in mixtures or under conditions not tested here.

### Olfactory preferences of parasitic nematodes reflect host specificity

The fact that *Str. stercoralis* responds to human-emitted odorants suggests that olfaction plays an important role in host finding. However, the extent to which *Str. stercoralis* or any other mammalian-parasitic nematode uses olfactory cues for host selection is not known. To gain insight into whether olfaction contributes to host choice, we compared the olfactory responses of *Str. stercoralis* to those of six other species: *Str. ratti, N. brasiliensis, Ha. contortus, He. bacteriophora, Ste. carpocapsae*, and *C. elegans*. We found that all species responded to a wide array of odorants, indicating that as is the case for EPNs [21], [22], even ambushers are capable of robust chemotaxis (Figure 3B and Figure S4). Moreover, each species exhibited a unique odor response profile, indicating that olfactory responses are species-specific even among closely related species such as *Str. stercoralis* and *Str. ratti* (Figure 3B). CO_2_ response varied greatly among species. Like *Str. stercoralis, Str. ratti* and *N. brasiliensis* were repelled by CO_2_ at high concentrations and neutral to CO_2_ at low concentrations (Figure 3B and Figure S4B–C). By contrast, *Ha. contortus* IJs, like EPN IJs and *C. elegans* dauers [21], [22], were attracted to CO_2_ (Figure 3B and Figure S4D). To confirm that the observed responses to odorants were olfactory rather than gustatory, we examined the responses of *Str. stercoralis* and *Str. ratti* to a subset of odorants in a modified chemotaxis assay in which odorants were placed on the plate lid rather than the plate surface. We found that attractive responses were still observed when the odorants were placed on the plate lid, although the response of *Str. stercoralis* to one odorant was slightly reduced (Figure S5). Thus, the observed behavioral responses are primarily olfactory, but in some cases may include a gustatory component.

The olfactory preferences of the passively ingested mammalian parasite, *Ha. contortus*, are consistent with its known ecology. *Ha. contortus* IJs migrate from the feces of their ruminant hosts to grass blades, where they are ingested by grazing ruminants [34]. The fact that 5% CO_2_, which approximates the concentration found in exhaled breath [35], was strongly attractive to *Ha. contortus* (Figure S4D) suggests that *Ha. contortus* may use exhaled CO_2_ to migrate toward the mouths of potential hosts. By contrast, *Ha. contortus* was repelled by many of the skin and sweat odorants tested (Figure 3B), consistent with a lack of attraction to mammalian skin. Of the few attractive odorants we identified for *Ha. contortus*, two – methyl myristate and myristic acid – are known constituents of cow and goat milk [36]–[38] and may be used by *Ha. contortus* to migrate toward cows and goats. To test whether *Ha. contortus* also responds to plant-emitted odorants, we examined responses to freshly cut grass. We found that *Ha. contortus* is attracted to the smell of grass, while *Str. stercoralis* and *Ste. carpocapsae* are not (Figure 3C). These results suggest that *Ha. contortus* uses CO_2_ in combination with other ruminant-emitted odorants and grass odorants to position itself for passive ingestion.

We then quantitatively compared odor response profiles across species, and found that species with similar hosts responded more similarly to odorants despite their phylogenetic distance (Figure 3D). For example, the distantly related rat parasites *Str. ratti* and *N. brasiliensis* responded similarly to odorants, as did the distantly related insect parasites *He. bacteriophora* and *Ste. carpocapsae*. The three skin-penetrating species responded more similarly to each other than to the other species tested, while the passively ingested mammalian parasite *Ha. contortus* responded very differently from all of the other species tested (Figure 3D). These results indicate that olfactory preferences reflect host specificity and infection mode rather than phylogeny, consistent with a key role for olfaction in host selection.

### Olfactory preferences of *Strongyloides* species are life stage-specific

**Figure 4:**
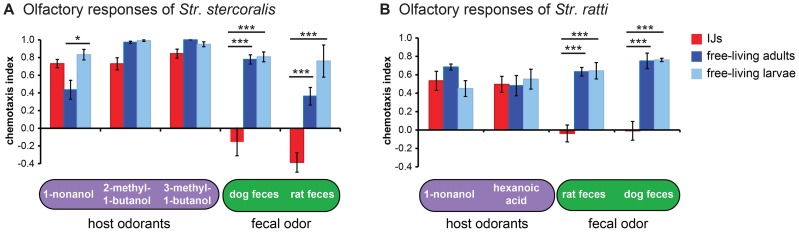
Olfactory responses of *Strongyloides* species vary across life stages. **A–B**. Responses of either *Str. stercoralis* (**A**) or *Str. ratti* (**B**) IJs, free-living adults, and free-living larvae to host odorants and fecal odor. *, *P*<0.05; ***, *P*<0.001, two-way ANOVA with Tukey's post-test. n = 4–12 trials for *Str. stercoralis* and n = 6–26 trials for *Str. ratti* for each condition. Error bars indicate SEM.

Skin-penetrating nematodes exit from hosts in feces as eggs or young larvae and subsequently develop into infective larvae outside the host. Thus, both infective and non-infective life stages are present in the environment (Figure 1B–C). This raises the question of whether host attraction is specific to the infective stage. We compared olfactory responses of free-living larvae, free-living adults, and IJs for both *Str. stercoralis* and *Str. ratti* in response to a subset of host odorants. We found that all three life stages were robustly attracted to host odorants, suggesting that host attraction is not downregulated in non-infective life stages (Figure 4). The free-living life stages of skin-penetrating worms are thought to reside primarily on host fecal matter, where they feed on bacteria present in the feces [39]. We therefore compared the responses of free-living larvae, free-living adults, and IJs to host feces. We found that responses differed dramatically across life stages: free-living larvae and adults were strongly attracted to feces, while IJs were neutral to host feces (Figure 4). Moreover, while *Str. ratti* IJs were neutral to both host and non-host feces, *Str. stercoralis* IJs were neutral to host feces but repelled by non-host feces (Figure 4).

Our results suggest a model in which all life stages are attracted to host skin odor, but strong attraction to host fecal odor by the free-living life stages causes them to remain on feces. Attraction to fecal odor is downregulated at the infective stage, enabling the IJs to migrate away from the feces in search of hosts. Repulsion of *Str. stercoralis* IJs from non-host feces may serve as an additional mechanism to prevent foraging in close proximity to non-hosts. To gain insight into the individual odorants that confer changes in sensitivity to feces, we examined responses to two components of fecal odor, skatole and indole [40]. We found that the free-living stages of *Str. ratti* were highly attracted to both skatole and indole, while the IJs were neutral to both odorants (Figure S6A). Thus, altered sensitivity to these odorants may contribute to the developmental change in the response to fecal odor. By contrast, *Str. stercoralis* IJs were more attracted to skatole than the free-living life stages and all three life stages were relatively unresponsive to indole (Figure S6B), suggesting that other as yet unidentified odorants mediate the sensitivity of *Str. stercoralis* to fecal odor.

### Implications for nematode control

*Str. stercoralis* infection is a worldwide cause of chronic morbidity and mortality. Current drugs used to treat nematode infections are inadequate for nematode control: some are toxic, drug resistance is a growing concern, and reinfection rates are high [41]. Our data suggest that *Str. stercoralis* IJs are fast-moving cruisers that actively search for hosts using a chemically diverse array of human-emitted odorants. The identification of odorants that attract or repel *Str. stercoralis* and other parasitic nematodes lays a foundation for the design of targeted traps or repellents, which could have broad implications for nematode control.

## Materials and Methods

### Ethics statement

Gerbils were used for host passage of *Str. stercoralis*, and rats were used for host passage of *Str. ratti* and *N. brasiliensis*. All protocols and procedures were approved by the UCLA Office of Animal Research Oversight (Protocol No. 2011-060-03B), which adheres to the AAALAC standards for laboratory animal use, and were in strict accordance with the *Guide for the Care and Use of Laboratory Animals*.

### Nematodes, vertebrate animals, and insects

*Strongyloides stercoralis* UPD strain and *Strongyloides ratti* ED321 strain were provided by Dr. James Lok (University of Pennsylvania). *Nippostrongylus brasiliensis* was provided by Dr. Edward Platzer (University of California, Riverside). *Haemonchus contortus* was provided by Dr. Adrian Wolstenholme and Mr. Bob Storey (University of Georgia). *Heterorhabditis bacteriophora* Oswego strain and *Steinernema glaseri* VS strain were provided by David Shapiro-Ilan (USDA). *Steinernema carpocapsae* were from the ALL strain [21], [22], [42]. *C. elegans* dauers were from the wild isolate CB4856 (“Hawaii”). Male Mongolian gerbils for culturing *Str. stercoralis* were obtained from Charles River Laboratories. Male or female Long-Evans or Sprague Dawley rats for culturing *Str. ratti* and *N. brasiliensis* were obtained either from Harlan Laboratories or second-hand from other investigators at UCLA through the UCLA Internal Animal Transfer supply system for surplus animals. *Galleria mellonella* larvae for culturing EPNs were obtained from American Cricket Ranch (Lakeside, CA).

### Maintenance of *Str. stercoralis*

*Str. stercoralis* was serially passaged in gerbils and maintained on fecal-charcoal plates. Inoculation of gerbils with *Str. stercoralis* was performed essentially as previously described [43]. Briefly, *Str. stercoralis* IJs were isolated from fecal-charcoal plates using a Baermann apparatus [43]. Each gerbil was subcutaneously injected with 2000 IJs in 200 µl sterile PBS. Gerbils became patent (as defined by the presence of nematodes in gerbil feces) on day 12 post-inoculation and remained patent for approximately 70 days. At 28 and 35 days post-inoculation, each gerbil received 2 mg methylprednisolone (Depo-Medrol, Pfizer) subcutaneously to induce an auto-infective cycle. To harvest infested feces, gerbils were housed overnight in cages containing a wire rack on the bottom of the cage. Fecal pellets fell below the rack onto damp cardboard and were collected the following morning. Feces were mixed with dH_2_O and autoclaved charcoal (bone char from Ebonex Corp., Cat # EBO.58BC.04) in an approximately 1∶1 ratio of charcoal to feces. The fecal-charcoal mixtures were poured into Petri dishes (10 cm diameter, 20 mm height) lined with wet filter paper, and were stored at 23°C until use. Nematodes used for behavioral analysis were isolated from fecal-charcoal plates using a Baermann apparatus [43] or from plate lids. To obtain free-living larvae (primarily post-parasitic L2s) for chemotaxis assays, nematodes were collected from fecal-charcoal plates after approximately 18 hrs. To obtain free-living adults for chemotaxis assays, nematodes were collected from fecal-charcoal plates after 48 hrs. To obtain IJs, nematodes were collected from fecal-charcoal plates starting at day 5 post-collection. IJs were used for behavioral assays within 2 weeks of fecal collection.

### Maintenance of *Str. ratti*

*Str. ratti* was serially passaged in rats and maintained on fecal-charcoal plates. Inoculation of rats with *Str. ratti* was performed essentially as previously described [44]. Briefly, *Str. ratti* IJs were isolated from fecal-charcoal plates using a Baermann apparatus. Each rat was subcutaneously injected with 700 IJs in 300 µl sterile PBS. Rats became patent on day 6 post-inoculation and remained patent for up to 28 days post-inoculation. To harvest infested feces, rats were housed overnight in cages containing a wire rack on the bottom of the cage. Fecal pellets fell below the rack onto damp cardboard and were collected the following morning. Fecal-charcoal plates were prepared as described above for *Str. stercoralis* and stored at 23°C until use. Nematodes used for behavioral analysis were isolated from fecal-charcoal plates using a Baermann apparatus [43] or from plate lids. Free-living larvae, adults, and IJs were obtained from fecal-charcoal plates as described above for *Str. stercoralis.*

### Maintenance of *N. brasiliensis*

*N. brasiliensis* was serially passaged in rats and maintained on fecal-charcoal plates. To inoculate rats, *N. brasiliensis* IJs were isolated from fecal-charcoal plates using a Baermann apparatus. Each rat was subcutaneously injected with 4000 IJs in 300 µl sterile PBS. Rats became patent on day 6 post-inoculation and remained patent for up to 14 days. Infested feces were collected as described above for *Str. ratti*. Fecal-charcoal plates were prepared as described above for *Str. stercoralis*, except that vermiculite (Fisher catalog # S17729) was added to the feces and charcoal in an approximately 1∶1∶1 ratio of vermiculite to charcoal to feces. Plates were stored at 23°C until use. In some cases, either Nystatin (Sigma catalog # N6261) at a concentration of 200 U/ml or Fungizone (Gibco catalog #15290-018) at a concentration of 1 µg/ml was added to the filter paper on the bottom of the plate to inhibit mold growth. Nematodes used for behavioral analysis were isolated from fecal-charcoal plates using a Baermann apparatus [43] or from plate lids. To obtain IJs, nematodes were collected from fecal-charcoal plates starting at day 7 post-collection. IJs were used for behavioral assays within 2 weeks of fecal collection.

### Maintenance of *Ha. contortus*

*Ha. contortus* was stored in dH_2_O at 8°C prior to use. IJs were tested within 6 months of collection. No differences in IJ movement or behavior were observed in freshly collected versus 6 month old IJs. IJ behavior declined after 6 months, so IJs older than 6 months were not tested.

### Maintenance of entomopathogenic nematodes (EPNs)

EPNs were cultured as previously described [21]. Briefly, 5 last instar *Galleria mellonella* larvae were placed in a 5 cm Petri dish with a 55 mm Whatman 1 filter paper acting as a pseudo-soil substrate in the bottom of the dish. Approximately 250 µl containing 500–1000 IJs suspended in water was evenly distributed on the filter paper. After 7–10 days the insect cadavers were placed on White traps [45]. Emerging IJs were collected from the White trap, rinsed 3 times with dH_2_O, and stored in dH_2_O until use. *Ste. carpocapsae* and *He. bacteriophora* were maintained at 25°C, while *Ste. glaseri* was maintained at room temperature. IJs were used for behavioral assays within 7 days of collection from the White trap.

### Maintenance of *C. elegans*

*C. elegans* was cultured on NGM plates seeded with *E. coli* OP50 according to standard methods [46]. Dauer larvae were collected from the lids of plates from which the nematodes had consumed all of the OP50 and stored in dH_2_O at room temperature prior to use. Dauer larvae were used for behavioral assays within 2 weeks of collection from plate lids.

### Motility assays

30–100 IJs were placed in the center of a chemotaxis plate [47]. IJs were allowed to distribute over the agar surface for 1 hr, after which the percentage of IJs in the outer zone (Zone 2) was determined. Zone 1 was a 4 cm diameter circle centered in the middle of the plate. Zone 2 consisted of the rest of the plate and included the edges of the plate, which acted as a trap since IJs that crawled onto the plate edge desiccated and could not return to the agar surface.

### Recording worm movement for automated tracking

Recordings of worm movement were obtained with an Olympus E-PM1 digital camera attached to a Leica S6 D microscope. To quantify unstimulated movement, 4–5 IJs were placed in the center of a chemotaxis plate [47] and allowed to acclimate for 10 min. 20 s recordings were then obtained. Worms that either did not move, that stopped moving during the recording, or that crawled off the assay plate during the recording were excluded from the analysis. To quantify movement before and after mechanical stimulation, IJs were placed on chemotaxis plates and allowed to acclimate for 10 min. prior to tracking. Baseline movement was recorded for approximately 15 s. The plate lid was then removed, the IJ was gently agitated using a worm pick, and post-agitation movement was recorded for approximately 30 s. 5 s recording clips directly following agitation were used to calculate the maximum speeds shown in Figure S1D, and 5 s recording clips directly preceding and following agitation were used to generate the sample tracks shown in Figure S1C. Maximum speeds were calculated in WormAnalyzer (see below) based on changes in worm position over a seven frame (or 0.23 second) window. To quantify movement following thermal stimulation, assays were performed in a 37°C warm room. Chemotaxis assay plates were kept in the warm room prior to use. Individual IJs were transported into the warm room, transferred to assay plates, and immediately recorded for 20 s. For the room temperature control, IJs were similarly transferred to assay plates and immediately recorded for 20 s. Locomotion was quantified using WormTracker and WormAnalyzer multi-worm tracker software (Miriam Goodman lab, Stanford University) [16]. The following WormTracker settings were adjusted from the default settings (designed for *C. elegans* adults) for analysis of IJ movement: min. single worm area = 20 pixels; max. size change by worm between successive frames = 250 pixels; shortest valid track = 30 frames; auto-thresholding correction factor = 0.001. To calculate turn frequencies, the following WormAnalyzer settings were adjusted from the default settings for analysis of IJ speed: sliding window for smoothing track data = 30 frames; minimum run duration for pirouette identification = 2.9 s for *Str. stercoralis*, 5.3 s for *Ste. glaseri*, and 6 s for all other species (to compensate for differences in speed among species). All turns were confirmed by visual observation of worm tracks; turns not confirmed by visual observation were not counted. For calculations of maximum displacement in Figure 2D, the distance between the worm's start point and the farthest point the worm reached during the 20 s recording was calculated in ImageJ.

### Nictation assays

Nictation was quantified on “micro-dirt” agar chips cast from polydimethylsiloxane (PDMS) molds as previously described [18], except that chips were made from 5% agar dissolved in dH_2_O and were incubated at 37°C for 2 hr and then room temperature for 1 hr before use. The micro-dirt chip consisted of agar with near-microscopic pillars covering its surface (pillar height of 25 µm with a radius of 25 µm and an interval between pillars of 25 µm), which allowed IJs to nictate on top of the pillars. For each assay, 3–10 IJs were transferred to the micro-dirt chip and allowed to acclimate on the chip for 10 min. Each IJ was then monitored for 2 min. An IJ was scored as “nictating” if it raised its head off the surface of the chip for a period of at least 5 s during the 2 min assay period. Nictation behavior was also tested on sand. Sand nictation assays were performed essentially as previously described [21], [48]. Sand (silicon dioxide, >230 mesh, CAS 60676-86-0) was distributed onto the surface of a chemotaxis plate using a sieve. IJs were transferred to the plate surface and allowed to acclimate for 10 min. Nictation behavior was then observed for two minutes. In all cases, nictation behavior on sand was consistent with nictation behavior on micro-dirt chips. In the case of *Ha. contortus*, we also tested for nictation on grass and vermiculite; no nictation was observed on any substrate tested. To test for nictation on grass, grass samples were collected from a lawn seeded with UC Verde Buffalo grass and perennial rye grass (the same lawn as for sample 1 below). The grass was cut into small chunks (∼2.5 mm×2.5 mm) and distributed onto the surface of a chemotaxis plate. IJs were transferred onto the plate surface or directly onto blades of grass, and nictation was scored after a 10 min. acclimation period. Nictation was also scored after 20, 30, or 60 min., or the next day. No nictation was observed with *Ha. contortus* at any time point.

### Odor chemotaxis assays

Odor chemotaxis assays were performed essentially as described [21], [22] (Figure S2). Assays were performed on chemotaxis assay plates [47]. Scoring regions consisted of 2 cm diameter circles on each side of the plate along the diameter with the center of the circle 1 cm from the edge of the plate, as well as the rectangular region extending from the edges of the circle to the edge of the plate. Either 2 µl (for mammalian-parasitic IJs) or 1 µl (for insect-parasitic IJs and *C. elegans* dauers) of 5% sodium azide was placed in the scoring region as anesthetic. 5 µl of odorant was then placed on the surface of the assay plate in the center of one scoring region, and 5 µl of control (paraffin oil, dH_2_O, or ethanol) was placed on the surface of the assay plate in the center of the other scoring region. Approximately 200 worms were placed in the center of the assay plate and left undisturbed on a vibration-reducing platform for 3 hours at room temperature. A chemotaxis index (CI) was then calculated as: CI = (# worms at odorant−# worms at control)/(# worms at odorant+control) (Figure S2). A positive CI indicates attraction; a negative CI indicates repulsion. A 3 hour assay duration was used because 3 hour assays were found to be most effective for EPNs [21], [49]. However, 1 hour assays were also performed with *Str. ratti*, and no significant differences were observed in 1 hour vs. 3 hour assays (Table S6). Two identical assays were always performed simultaneously with the odor gradient in opposite directions on the two plates to control for directional bias due to room vibration; assays were discarded if the difference in the CIs for the two plates was ≥0.9 or if fewer than 7 worms moved into the scoring regions on one or both of the plates. Liquid odorants were tested undiluted unless otherwise indicated. Solid odorants were prepared as follows: 1-dodecanol, methyl palmitate, and methyl myristate were diluted 0.05 g in 2.5 ml paraffin oil; palmitic acid was diluted 10 g in 200 ml ethanol; myristic acid, skatole, and indole were diluted 0.05 g in 2.5 ml ethanol; and L-lactic acid was diluted 0.05 g in 2.5 ml dH_2_O. Ammonia was purchased as a 2 M solution in ethanol. Solid odorants were tested at these concentrations unless otherwise indicated. For assays in which odorants were placed on the plate lid rather than the plate surface (Figure S5), filter paper squares of approximately 0.5 cm in width were attached to the plate lid using double-stick tape. Odorant or control was then pipetted onto the filter paper, and chemotaxis was examined as described above.

### CO_2_ chemotaxis assays

CO_2_ chemotaxis assays were performed essentially as described [21], [22]. Assays were performed on chemotaxis assay plates [47], and scoring regions were as described above for odor chemotaxis assays (Figure S2). Gases were delivered at a rate of 0.5 ml/min through holes in the plate lids from gastight syringes filled with either a CO_2_ mixture containing the test concentration of CO_2_, 10% O_2_, and the balance N_2_, or a control air mixture containing 10% O_2_ and 90% N_2_. Certified gas mixtures were obtained from Air Liquide or Airgas. Assays were performed and scored as described above for odor chemotaxis assays, except that the assay duration was 1 hour.

### Grass chemotaxis assays

Fresh grass samples were collected from the campus of the University of California, Los Angeles. Sample 1 was collected from a lawn seeded with UC Verde Buffalo grass and perennial rye grass, and sample 2 was collected from a lawn seeded with a custom blend of annual ryegrass, *Festuca*, Bonsai dwarf fescue, Bermuda grass, and bluegrass. 200 µl of dH_2_O was added to 0.1 g grass. Grass was then ground in a small weigh boat, and 5 µl of the grass suspension was used in a chemotaxis assay with 5 µl dH_2_O as a control. Grass was either used immediately for chemotaxis assays or stored at 4°C for no more than 3 days.

### Fecal chemotaxis assays

Uninfected rat or dog feces was collected from animals in the UCLA vivarium. Responses to feces were tested using a modified chemotaxis assay in which feces was placed on the plate lid rather than the plate surface. Filter paper squares of approximately 0.5 cm in width were attached to the plate lid using double-stick tape. Fecal matter was moistened with dH_2_O, smeared onto filter paper, and tested in a chemotaxis assay as described above for odor chemotaxis assays. We note that similar attraction to feces was observed when filter paper with feces was tested against filter paper with dH_2_O, and no attraction was observed to wet filter paper when wet filter paper was tested against dry filter paper (data not shown).

### Data analysis

Statistical analysis was performed using either GraphPad Instat, GraphPad Prism, or PAST [50]. The heatmap was generated using Heatmap Builder [51].

## Supporting Information

Figure S1 **IJ movement across species.** **A**. Unstimulated mean speeds of IJs. IJ speed varies among species (*P*<0.0001, Kruskal-Wallis test). *Str. stercoralis* crawled significantly faster than the other species tested (*P*<0.05, Dunn's post-test). Statistical analysis is shown in Table S2. n = 20–31 IJs for each species. **B**. Unstimulated turn frequencies of IJs in turns/s. Turn frequency varied among species (*P*<0.0001, Kruskal-Wallis test with Dunn's post-test) but did not correlate with speed (R^2^ = 0.22 and *P* = 0.28, linear correlation analysis). Statistical analysis is shown in Table S3. n = 20–31 IJs for each species. **C**. Representative tracks of *Ste. carpocapsae, Str. ratti*, and *Str. stercoralis* before and after mechanical stimulation. Recordings show 5 s of pre-stimulation movement and 5 s of post-stimulation movement. Red lines indicate the timing of the mechanical stimulation; red dot indicates the maximum speed attained during each recording; blue lines indicate the mean unstimulated speed for each species. **D**. Unstimulated vs. mechanically stimulated maximum speeds of IJs. ***, *P*<0.001; **, *P*<0.01, unpaired t test or Mann-Whitney test. n = 20–31 trials for unstimulated speed, 5–10 trials for stimulated speed. Maximum speed was used for this analysis since the species differed in how quickly they returned to basal speed following mechanical stimulation. For all graphs, error bars indicate SEM.
(PDF)

Figure S2 **Chemotaxis assay for IJs.** Odorant is placed on one side of the plate and control is placed on the other side (black dots). IJs are placed in the center of the plate and allowed to distribute in the odor gradient for 3 hr. The number of IJs in each scoring region is then counted, and a chemotaxis index is calculated as shown (right). The chemotaxis index ranges from +1 to −1, with a positive chemotaxis index indicating attraction and a negative chemotaxis index indicating repulsion. Red bar = 1 cm.
(PDF)

Figure S3 **Responses of** ***Str. stercoralis*** **to diluent controls.** Responses of *Str. stercoralis* to paraffin oil vs. paraffin oil, water vs. water, and ethanol vs. ethanol in a chemotaxis assay. The diluents did not elicit responses from *Str. stercoralis*, resulting in an equal distribution of IJs on both sides of the assay plate. n = 10–12 trials for each condition. Error bars indicate SEM.
(PDF)

Figure S4 **Responses to odorants across concentrations.** **A–D**. Responses of *Str. stercoralis* (**A**), *Str. ratti* (**B**), *N. brasiliensis* (**C**), and *Ha. contortus* (**D**) to increasing concentrations of odorants in a chemotaxis assay. n = 6–21 trials for each species-odorant combination.
(PDF)

Figure S5 **Responses to odorants are primarily olfactory rather than gustatory.** **A**. Responses of *Str. stercoralis* in a standard chemotaxis assay where odorants are placed on the plate surface vs. a modified chemotaxis assay where odorants are placed on the plate lid. Responses to 3-heptanol and 1-nonanol were not significantly different, while the response to 3-methyl-1-butanol was slightly reduced. **, *P*<0.01, two-way ANOVA with Bonferroni post-test. n = 6–12 trials for each condition. **B**. Responses of *Str. ratti* were not significantly different in the lid assay vs. the plate assay (*P*>0.05, two-way ANOVA). n = 6–16 trials for each condition. For all graphs, error bars indicate SEM.
(PDF)

Figure S6 **Responses of** ***Strongyloides*** **species to selected fecal odorants.** **A**. Responses of *Str. ratti* to skatole and indole across life stages. Both odorants were neutral for IJs but attractive for free-living larvae and adults. **, *P*<0.01; ***, *P*<0.001, two-way ANOVA with Tukey's post-test. n = 8–13 trials for each odorant. **B**. Responses of *Str. stercoralis* to skatole and indole. *, *P*<0.05, two-way ANOVA with Tukey's post-test. For all graphs, error bars indicate SEM.
(PDF)

Table S1 **Results of statistical analysis comparing motility across species.** *P* values were determined by one-way ANOVA with Tukey-Kramer post-test. ***, *P*<0.001; **, *P*<0.01; *, *P*<0.05; ns = not significant. Data are from Figure 2A.
(DOCX)

Table S2 **Results of statistical analysis comparing unstimulated mean speeds across species.** *P* values were determined by Kruskal-Wallis test with Dunn's post-test. ***, *P*<0.001; **, *P*<0.01; *, *P*<0.05; ns = not significant. Data are from Figure S1A.
(DOCX)

Table S3 **Results of statistical analysis comparing unstimulated turn frequencies across species.** *P* values were determined by Kruskal-Wallis test with Dunn's post-test. ***, *P*<0.001; **, *P*<0.01; *, *P*<0.05; ns = not significant. Data are from Figure S1B.
(DOCX)

Table S4 **Results of statistical analysis comparing nictation frequencies across species.** *P* values were determined by chi-square analysis with Bonferroni corrections for multiple comparisons. **, *P*<0.01; *, *P*<0.05; ns = not significant. Data are from Figure 2E.
(DOCX)

Table S5 **Mammalian-derived odorants tested.** Sources listed are not exhaustive.
(DOCX)

Table S6 **Comparison of 1 hour and 3 hour chemotaxis assays with** ***Str. ratti*** **.** Results from 1 hour and 3 hour assays were not significantly different (two-way ANOVA).
(DOCX)

Movie S1 **Mechanical stimulation of a** ***Str. ratti*** **IJ.**
(MOV)

Movie S2 **Mechanical stimulation of a** ***Str. stercoralis*** **IJ.**
(MOV)

Movie S3 **Nictation of an** ***N. brasiliensis*** **IJ.**
(MOV)

Movie S4 **Nictation of an** ***Str. ratti*** **IJ.**
(MOV)
