# Supplementary material for: Diverse Host-Seeking Behaviors of Skin-Penetrating Nematodes
Source: PLoS Pathog. 2014 Aug 14;10(8):e1004305. doi: 10.1371/journal.ppat.1004305 (PMC4133384; doi:10.1371/journal.ppat.1004305)
Supplement: Figure S1 — IJ movement across species. A. Unstimulated mean speeds of IJs. IJ speed varies among species (P<0.0001, Kruskal-Wallis test). Str. stercoralis crawled significantly faster than the other species tested (P<0.05, Dunn's post-test). Statistical analysis is shown in Table S2. n = 20–31 IJs for each species. B. Unstimulated turn frequencies of IJs in turns/s. Turn frequency varied among species (P<0.0001, Kruskal-Wallis test with Dunn's post-test) but did not correlate with speed (R2 = 0.22 and P = 0.28, linear correlation analysis). Statistical analysis is shown in Table S3. n = 20–31 IJs for each species. C. Representative tracks of Ste. carpocapsae, Str. ratti, and Str. stercoralis before and after mechanical stimulation. Recordings show 5 s of pre-stimulation movement and 5 s of post-stimulation movement. Red lines indicate the timing of the mechanical stimulation; red dot indicates the maximum speed attained during each recording; blue lines indicate the mean unstimulated speed for each species. D. Unstimulated vs. mechanically stimulated maximum speeds of IJs. ***, P<0.001; **, P<0.01, unpaired t test or Mann-Whitney test. n = 20–31 trials for unstimulated speed, 5–10 trials for stimulated speed. Maximum speed was used for this analysis since the species differed in how quickly they returned to basal speed following mechanical stimulation. For all graphs, error bars indicate SEM. (PDF) [file ppat.1004305.s001.pdf]

Figure S1

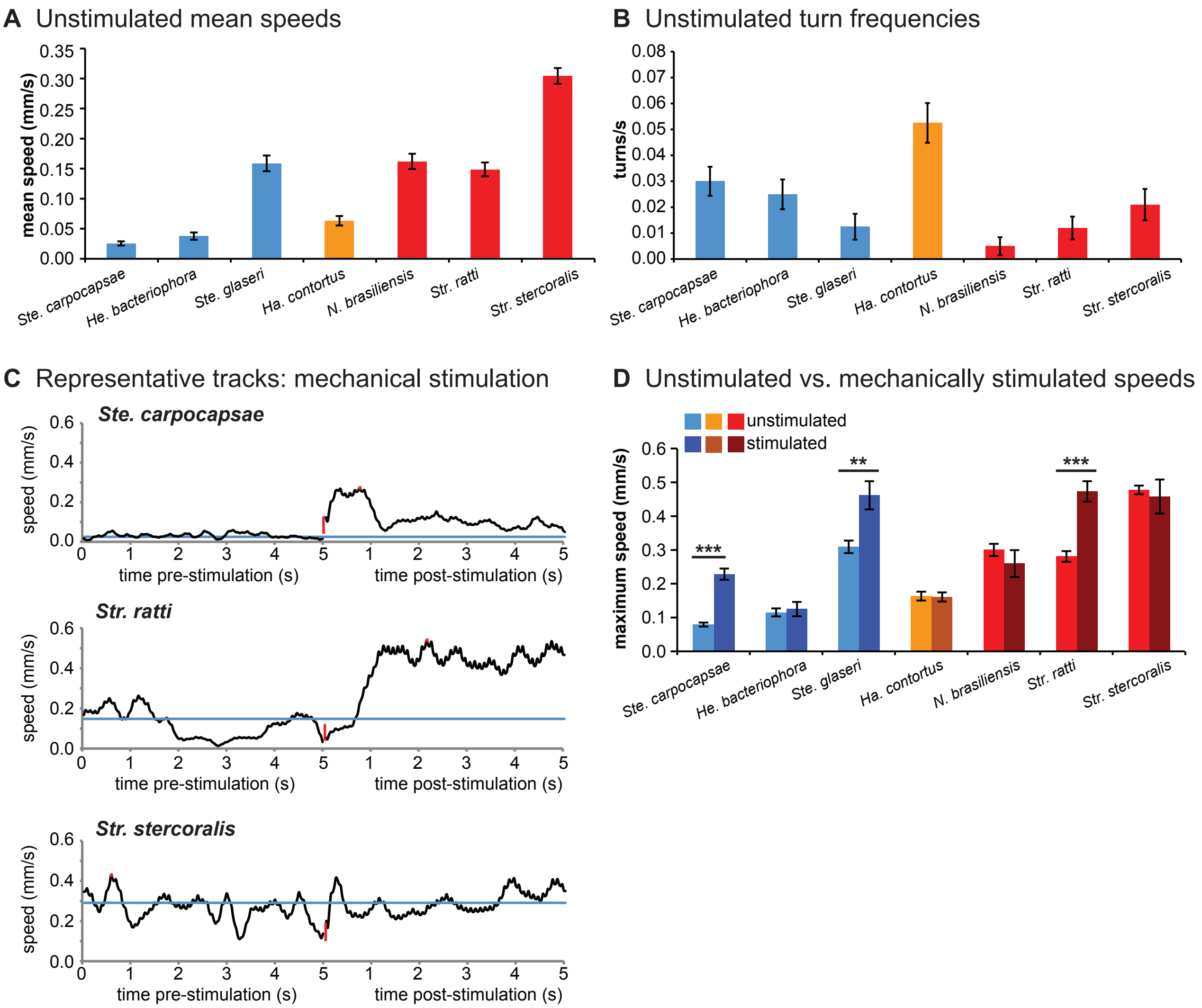

A Unstimulated mean speeds
mean speed (mm/s)
0.35
0.30
0.25
0.20
0.15
0.10
0.05
0.00
Ste. carpocapsae
He. bacteriophora
Ste. glaseri
Ha. contortus
N. brasiliensis
Str. ratti
Str. stercoralis
B Unstimulated turn frequencies
turns/s
0.08
0.07
0.06
0.05
0.04
0.03
0.02
0.01
0.00
Ste. carpocapsae
He. bacteriophora
Ste. glaseri
Ha. contortus
N. brasiliensis
Str. ratti
Str. stercoralis
C Representative tracks: mechanical stimulation
Ste. carpocapsae
speed (mm/s)
0.6
0.4
0.2
0.0
0 1 2 3 4 5 1 2 3 4 5
time pre-stimulation (s)
time post-stimulation (s)
Str. ratti
speed (mm/s)
0.6
0.4
0.2
0.0
0 1 2 3 4 5 1 2 3 4 5
time pre-stimulation (s)
time post-stimulation (s)
Str. stercoralis
speed (mm/s)
0.6
0.4
0.2
0.0
0 1 2 3 4 5 1 2 3 4 5
time pre-stimulation (s)
time post-stimulation (s)
D Unstimulated vs. mechanically stimulated speeds
unstimulated
stimulated
maximum speed (mm/s)
0.6
0.5
0.4
0.3
0.2
0.1
0.0
***
**
***
Ste. carpocapsae
He. bacteriophora
Ste. glaseri
Ha. contortus
N. brasiliensis
Str. ratti
Str. stercoralis
