# Supplementary material for: Diverse Host-Seeking Behaviors of Skin-Penetrating Nematodes
Source: PLoS Pathog. 2014 Aug 14;10(8):e1004305. doi: 10.1371/journal.ppat.1004305 (PMC4133384; doi:10.1371/journal.ppat.1004305)
Supplement: Figure S2 — Chemotaxis assay for IJs. Odorant is placed on one side of the plate and control is placed on the other side (black dots). IJs are placed in the center of the plate and allowed to distribute in the odor gradient for 3 hr. The number of IJs in each scoring region is then counted, and a chemotaxis index is calculated as shown (right). The chemotaxis index ranges from +1 to −1, with a positive chemotaxis index indicating attraction and a negative chemotaxis index indicating repulsion. Red bar = 1 cm. (PDF) [file ppat.1004305.s002.pdf]

Figure S2

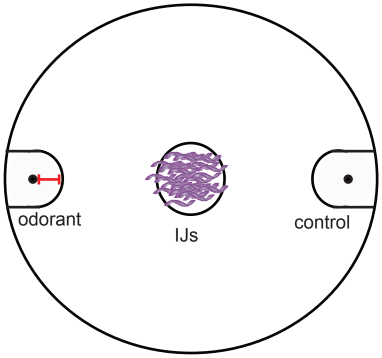

$$\text{chemotaxis index} = \frac{(\text{\# of worms at odorant}) - (\text{\# of worms at control})}{(\text{\# of worms at odorant}) + (\text{\# of worms at control})}$$
