# Supplementary figures and images for: Diverse Host-Seeking Behaviors of Skin-Penetrating Nematodes
Source: PLoS Pathog. 2014 Aug 14;10(8):e1004305. doi: 10.1371/journal.ppat.1004305 (PMC4133384; doi:10.1371/journal.ppat.1004305)

Figure S3

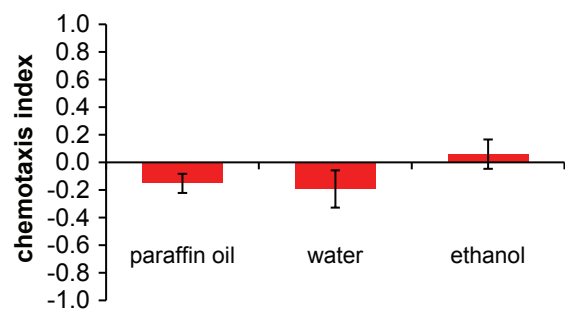

Supplement: Figure S3 — Responses of Str. stercoralis to diluent controls. Responses of Str. stercoralis to paraffin oil vs. paraffin oil, water vs. water, and ethanol vs. ethanol in a chemotaxis assay. The diluents did not elicit responses from Str. stercoralis, resulting in an equal distribution of IJs on both sides of the assay plate. n = 10–12 trials for each condition. Error bars indicate SEM. (PDF) [file ppat.1004305.s003.pdf]

Figure S5

**A** *Str. stercoralis* plate vs. lid assays

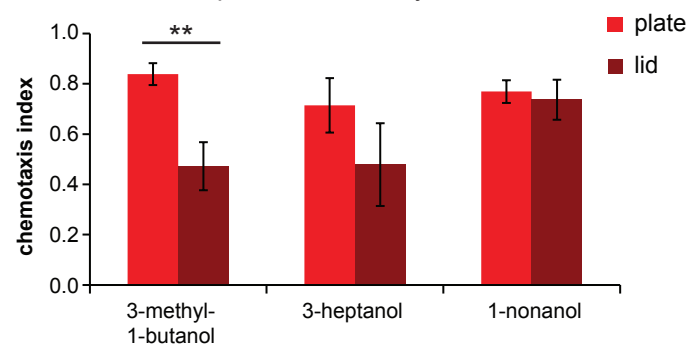

**B** *Str. ratti* plate vs. lid assays

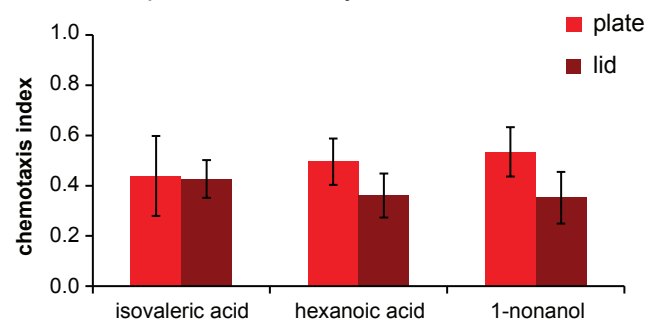

Supplement: Figure S5 — Responses to odorants are primarily olfactory rather than gustatory. A. Responses of Str. stercoralis in a standard chemotaxis assay where odorants are placed on the plate surface vs. a modified chemotaxis assay where odorants are placed on the plate lid. Responses to 3-heptanol and 1-nonanol were not significantly different, while the response to 3-methyl-1-butanol was slightly reduced. **, P<0.01, two-way ANOVA with Bonferroni post-test. n = 6–12 trials for each condition. B. Responses of Str. ratti were not significantly different in the lid assay vs. the plate assay (P>0.05, two-way ANOVA). n = 6–16 trials for each condition. For all graphs, error bars indicate SEM. (PDF) [file ppat.1004305.s005.pdf]

Figure S6

**A** Olfactory responses of *Str. ratti*

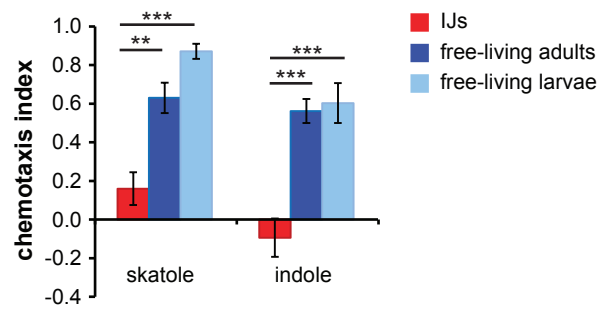

**B** Olfactory responses of *Str. stercoralis*

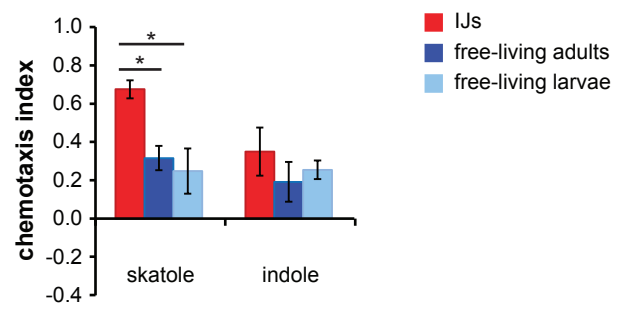

Supplement: Figure S6 — Responses of Strongyloides species to selected fecal odorants. A. Responses of Str. ratti to skatole and indole across life stages. Both odorants were neutral for IJs but attractive for free-living larvae and adults. **, P<0.01; ***, P<0.001, two-way ANOVA with Tukey's post-test. n = 8–13 trials for each odorant. B. Responses of Str. stercoralis to skatole and indole. *, P<0.05, two-way ANOVA with Tukey's post-test. For all graphs, error bars indicate SEM. (PDF) [file ppat.1004305.s006.pdf]
