# Supplementary material for: Diverse Host-Seeking Behaviors of Skin-Penetrating Nematodes
Source: PLoS Pathog. 2014 Aug 14;10(8):e1004305. doi: 10.1371/journal.ppat.1004305 (PMC4133384; doi:10.1371/journal.ppat.1004305)
Supplement: Figure S4 — Responses to odorants across concentrations. A–D. Responses of Str. stercoralis (A), Str. ratti (B), N. brasiliensis (C), and Ha. contortus (D) to increasing concentrations of odorants in a chemotaxis assay. n = 6–21 trials for each species-odorant combination. (PDF) [file ppat.1004305.s004.pdf]

Figure S4

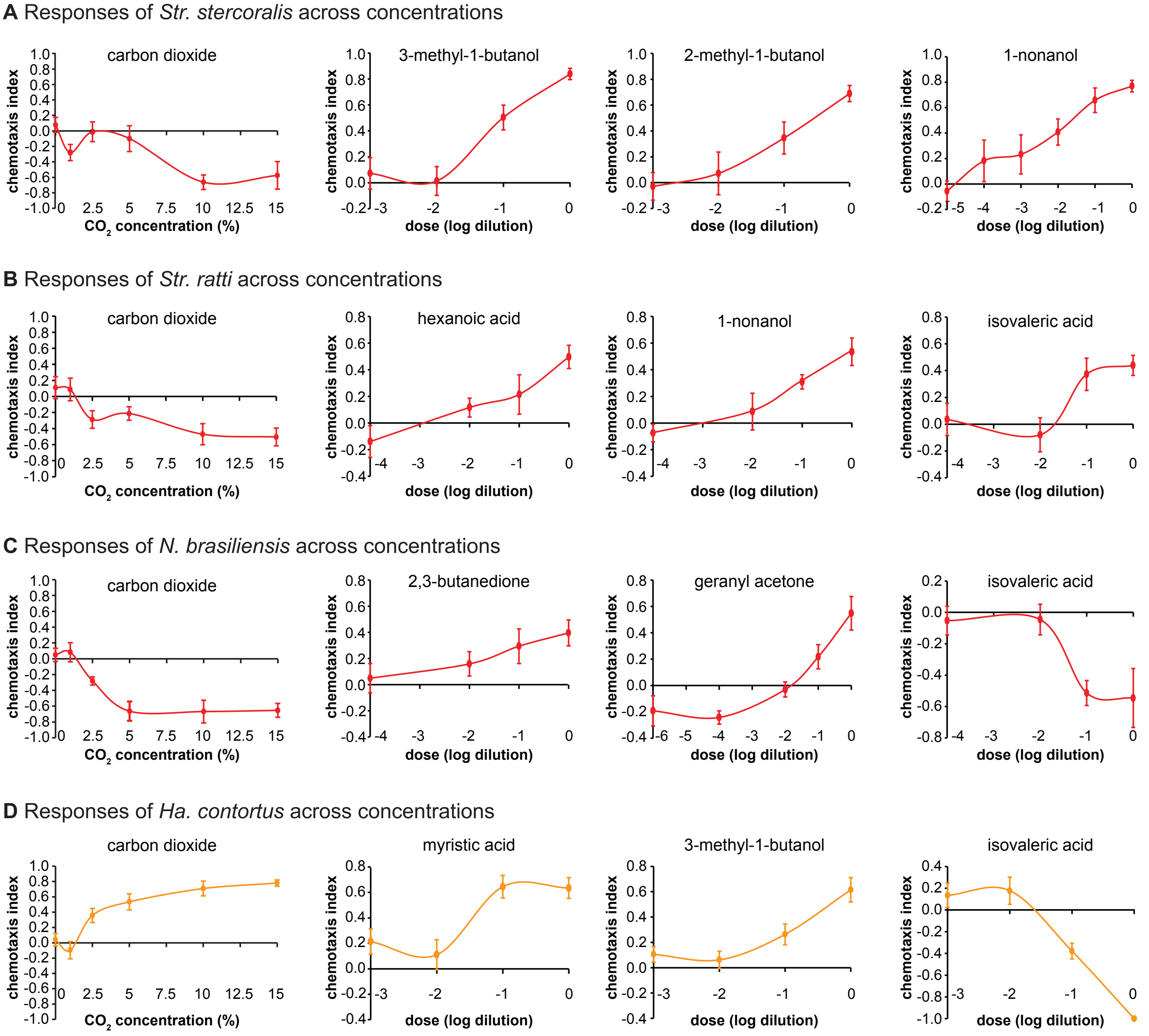
A Responses of *Str. stercoralis* across concentrations
carbon dioxide
3-methyl-1-butanol
2-methyl-1-butanol
1-nonanol
B Responses of *Str. ratti* across concentrations
carbon dioxide
hexanoic acid
1-nonanol
isovaleric acid
C Responses of *N. brasiliensis* across concentrations
carbon dioxide
2,3-butanedione
geranyl acetone
isovaleric acid
D Responses of *Ha. contortus* across concentrations
carbon dioxide
myristic acid
3-methyl-1-butanol
isovaleric acid
chemotaxis index
CO_2 concentration (%)
dose (log dilution)
