# Supplementary material for: Diverse Host-Seeking Behaviors of Skin-Penetrating Nematodes
Source: PLoS Pathog. 2014 Aug 14;10(8):e1004305. doi: 10.1371/journal.ppat.1004305 (PMC4133384; doi:10.1371/journal.ppat.1004305)
Supplement: Table S4 — Results of statistical analysis comparing nictation frequencies across species. P values were determined by chi-square analysis with Bonferroni corrections for multiple comparisons. **, P<0.01; *, P<0.05; ns = not significant. Data are from Figure 2E. (DOCX) [file ppat.1004305.s010.docx]

**Table S4. Results of statistical analysis comparing nictation frequencies across species.** *P* values were determined by chi-square analysis with Bonferroni corrections for multiple comparisons. **, *P*<0.01; *, *P*<0.05; ns = not significant. Data are from Figure 2E.

| **Pairwise species comparisons** | ***P* value** |
| --- | --- |
| *Strongyloides stercoralis* vs. *Strongyloides ratti* | ns |
| *Strongyloides stercoralis* vs. *Nippostrongylus brasiliensis* | ** |
| *Strongyloides stercoralis* vs. *Haemonchus contortus* | ns |
| *Strongyloides stercoralis* vs. *Heterorhabditis bacteriophora* | ns |
| *Strongyloides stercoralis* vs. *Steinernema carpocapsae* | ns |
| *Strongyloides ratti* vs. *Nippostrongylus brasiliensis* | ** |
| *Strongyloides ratti* vs. *Haemonchus contortus* | * |
| *Strongyloides ratti* vs. *Heterorhabditis bacteriophora* | ns |
| *Strongyloides ratti* vs. *Steinernema carpocapsae* | ns |
| *Nippostrongylus brasiliensis* vs. *Haemonchus contortus* | ** |
| *Nippostrongylus brasiliensis* vs. *Heterorhabditis bacteriophora* | ** |
| *Nippostrongylus brasiliensis* vs. *Steinernema carpocapsae* | ns |
| *Haemonchus contortus* vs. *Heterorhabditis bacteriophora* | ns |
| *Haemonchus contortus* vs. *Steinernema carpocapsae* | ** |
| *Steinernema carpocapsae* vs. *Heterorhabditis bacteriophora* | * |
