# Supplementary material for: Diverse Host-Seeking Behaviors of Skin-Penetrating Nematodes
Source: PLoS Pathog. 2014 Aug 14;10(8):e1004305. doi: 10.1371/journal.ppat.1004305 (PMC4133384; doi:10.1371/journal.ppat.1004305)
Supplement: Table S5 — Mammalian-derived odorants tested. Sources listed are not exhaustive. (DOCX) [file ppat.1004305.s011.docx]

**Table S5. Mammalian-derived odorants tested.** Sources listed are not exhaustive.

| **Odorant** | **Class** | **Source** | **References** |
| --- | --- | --- | --- |
| ethanol | alcohol | human: sweat, feces | [[1](#_ENREF_1),[2](#_ENREF_2)] |
| 1-heptanol | alcohol | human: sweat, hair, scalp, feces  dog: feces | [[1-4](#_ENREF_1)] |
| 1-octanol | alcohol | human: sweat, hair, scalp, feces | [[1-3](#_ENREF_1)] |
| 1-nonanol | alcohol | human: sweat, hand, hair, scalp, feces | [[1-3](#_ENREF_1)] |
| 1-dodecanol | alcohol | human: sweat, feces | [[1](#_ENREF_1),[2](#_ENREF_2)] |
| 2-methyl-1-butanol | alcohol | human: sweat, skin microbiota | [[1](#_ENREF_1),[5](#_ENREF_5)] |
| 3-methyl-1-butanol | alcohol | human: sweat, skin microbiota, feces | [[1](#_ENREF_1),[2](#_ENREF_2),[5](#_ENREF_5)] |
| farnesol | alcohol | human: sebum  dog: feces  rat: preputial gland | [[4](#_ENREF_4),[6](#_ENREF_6),[7](#_ENREF_7)] |
| methyl myristate | acetate ester | human: armpit | [[8](#_ENREF_8)] |
| methyl palmitate | acetate ester | human: hand, armpit, foot | [[8-12](#_ENREF_8)] |
| 2-methylpropanal | aldehyde | human: urine, feces | [[2](#_ENREF_2),[13](#_ENREF_13),[14](#_ENREF_14)] |
| 2-butanone | ketone | human: hand, urine, blood, breath, feces | [[2](#_ENREF_2),[13-16](#_ENREF_13)] |
| 2-hexanone | ketone | human: urine, blood, breath, hair, scalp, feces  dog: hair | [[2](#_ENREF_2),[3](#_ENREF_3),[14](#_ENREF_14),[16-18](#_ENREF_16)] |
| 2-heptanone | ketone | human: urine, blood, breath, hair, scalp, feces  dog: feces  rat: urine | [[2-4](#_ENREF_2),[13](#_ENREF_13),[14](#_ENREF_14),[16](#_ENREF_16),[19](#_ENREF_19)] |
| 2,3-butanedione | ketone | human: skin microbiota, urine, blood, breath, feces | [[2](#_ENREF_2),[5](#_ENREF_5),[14](#_ENREF_14),[16](#_ENREF_16)] |
| 6-methyl-5-hepten-2-one | ketone | human: hand, armpit, back or arm, sweat, forearm, foot, feces | [[1](#_ENREF_1),[2](#_ENREF_2),[8](#_ENREF_8),[11](#_ENREF_11),[12](#_ENREF_12),[15](#_ENREF_15),[20-22](#_ENREF_20)] |
| geranyl acetone | ketone | human: sweat, back/arm, forearm, hand, foot  dog: feces | [[1](#_ENREF_1),[4](#_ENREF_4),[12](#_ENREF_12),[20-22](#_ENREF_20)] |
| acetic acid | acid | human: foot, hand, sweat, back/arm, hair, scalp, breath | [[1](#_ENREF_1),[3](#_ENREF_3),[15](#_ENREF_15),[20](#_ENREF_20),[23-25](#_ENREF_23)] |
| octanoic acid | acid | human: foot, hand, face, trunk, armpit, feces | [[2](#_ENREF_2),[10-12](#_ENREF_10),[15](#_ENREF_15),[22](#_ENREF_22),[23](#_ENREF_23),[26](#_ENREF_26),[27](#_ENREF_27)] |
| nonanoic acid | acid | human: foot, hand, face, trunk, armpit | [[10-12](#_ENREF_10),[15](#_ENREF_15),[26](#_ENREF_26),[28](#_ENREF_28)] |
| myristic acid | acid | human: hand, face, trunk, armpit, back/arm  dog: feces  ruminant: milk  rat: preputial gland, sebum | [[4](#_ENREF_4),[8](#_ENREF_8),[9](#_ENREF_9),[15](#_ENREF_15),[19](#_ENREF_19),[20](#_ENREF_20),[26](#_ENREF_26),[29](#_ENREF_29),[30](#_ENREF_30)] |
| palmitic acid | acid | human: hand, face, trunk, back/arm, urine  dog: feces  rat: preputial gland, sebum | [[4](#_ENREF_4),[9](#_ENREF_9),[15](#_ENREF_15),[17](#_ENREF_17),[19](#_ENREF_19),[20](#_ENREF_20),[26](#_ENREF_26),[30](#_ENREF_30)] |
| stearic acid | acid | human: hand, face, trunk, urine  dog: feces  rat: preputial gland, sebum | [[4](#_ENREF_4),[9](#_ENREF_9),[15](#_ENREF_15),[17](#_ENREF_17),[19](#_ENREF_19),[26](#_ENREF_26),[30](#_ENREF_30)] |
| isovaleric acid | acid | human: foot, back/arm, sweat | [[1](#_ENREF_1),[20](#_ENREF_20),[23](#_ENREF_23),[24](#_ENREF_24)] |
| L-lactic acid | acid | human: hand, back, back/arm, sweat, urine | [[9](#_ENREF_9),[15](#_ENREF_15),[17](#_ENREF_17),[20](#_ENREF_20),[31](#_ENREF_31),[32](#_ENREF_32)] |
| 7-octenoic acid | acid | human: armpit, sweat | [[10](#_ENREF_10),[27](#_ENREF_27),[33](#_ENREF_33)] |
| benzaldehyde | aromatic | human: hand, armpit, back/arm, urine, arm, foot, blood, breath, feces  dog: hair, feces  rat: preputial gland, urine | [[2](#_ENREF_2),[4](#_ENREF_4),[8](#_ENREF_8),[11](#_ENREF_11),[12](#_ENREF_12),[14-16](#_ENREF_14),[18](#_ENREF_18),[19](#_ENREF_19),[34](#_ENREF_34)] |
| p-cresol | aromatic | human: back or arm, face, trunk, feces, urine  rat: urine | [[2](#_ENREF_2),[17](#_ENREF_17),[19](#_ENREF_19),[20](#_ENREF_20),[26](#_ENREF_26)] |
| indole | aromatic heterocyclic | human: feces, hand, sweat, forearm  dog: feces  rat: preputial gland | [[1](#_ENREF_1),[2](#_ENREF_2),[4](#_ENREF_4),[15](#_ENREF_15),[19](#_ENREF_19),[21](#_ENREF_21),[35](#_ENREF_35)] |
| skatole | aromatic heterocyclic | human: feces | [[35](#_ENREF_35)] |
| ammonia | base | human: skin microbiota, sweat | [[31](#_ENREF_31),[36](#_ENREF_36)] |
| squalane | hydrocarbon | human: sebum | [[37](#_ENREF_37)] |
| (+)-3-carene | monoterpene | human: blood, breath, feces | [[2](#_ENREF_2),[16](#_ENREF_16)] |

1. Meijerink J, Braks MAH, Brack AA, Adam W, Dekker T, et al. (2000) Identification of olfactory stimulants for *Anopheles gambiae* from human sweat samples. J Chem Ecol 26: 1367-1382.

2. Garner CE, Smith S, de Lacy Costello B, White P, Spencer R, et al. (2007) Volatile organic compounds from feces and their potential for diagnosis of gastrointestinal disease. Faseb J 21: 1675-1688.

3. Goetz N, Kaba G, Good D, Hussler G, Bore P (1988) Detection and identification of volatile compounds evolved from human hair and scalp using headspace gas chromatography. J Soc Cosmet Chem 39: 1-13.

4. Arnould C, Malosse C, Signoret J-P, Descoins C (1998) Which chemical constituents from dog feces are involved in its food repellent effect in sheep? J Chem Ecol 24: 559-576.

5. Verhulst NO, Beijleveld H, Knols BGJ, Takken W, Schraa G, et al. (2009) Cultured skin microbiota attracts malaria mosquitoes. Malaria J 8: 302.

6. Ponmanickam P, Palanivelu K, Govindaraj S, Baburajendran R, Habara Y, et al. (2010) Identification of testosterone-dependent volatile compounds and proteins in the preputial gland of rat *Rattus norvegicus*. Gen Comp Endocrinol 167: 35-43.

7. Nicolaides N (1965) Skin lipids. IV. Biochemistry and function. J Am Oil Chem Soc 42: 708-712.

8. Curran AM, Rabin SI, Prada PA, Furton KG (2005) Comparison of the volatile organic compounds present in human odor using SPME-GC/MS. J Chem Ecol 31: 1607-1619.

9. Bernier UR, Booth MM, Yost RA (1999) Analysis of human skin emanations by gas chromatography/mass spectrometry. 1. Thermal desorption of attractants for the yellow fever mosquito (*Aedes aegypti*) from handled glass beads. Anal Chem 71: 1-7.

10. Zeng XN, Leyden JJ, Spielman AI, Preti G (1996) Analysis of characteristic human female axillary odors: qualitative comparison to males. J Chem Ecol 22: 237-257.

11. Prada PA, Curran AM, Furton KG (2011) The evaluation of human hand odor volatiles on various textiles: a comparison between contact and noncontact sampling methods. J Forensic Sci 56: 866-881.

12. Dormont L, Bessiere J-M, McKey D, Cohuet A (2013) New methods for field collection of human skin volatiles and perspectives for their application in the chemical ecology of human-pathogen-vector interactions. J Exp Biol 216: 2783-2788.

13. Rudnicka J, Mochalski P, Agapiou A, Statheropoulos M, Amann A, et al. (2010) Application of ion mobility spectrometry for the detection of human urine. Anal Bioanal Chem 398: 2031–2038.

14. Matsumoto KE, Partridge DH, Robinson AB, Pauling L, Flath RA, et al. (1973) The identification of volatile compounds in human urine. J Chromatogr A 85: 31-34.

15. Bernier UR, Kline DL, Barnard DR, Schreck CE, Yost RA (2000) Analysis of human skin emanations by gas chromatography/mass spectrometry. 2. Identification of volatile compounds that are candidate attractants for the yellow fever mosquito (*Aedes aegypti*). Anal Chem 72: 747-756.

16. Mochalski P, King J, Klieber M, Unterkofler K, Hinterhuber H, et al. (2013) Blood and breath levels of selected volatile organic compounds in healthy volunteers. Analyst 138: 2134-2145.

17. Bouatra S, Aziat F, Mandal R, Guo AC, Wilson MR, et al. (2013) The human urine metabolome. PLoS ONE 8: e73076.

18. de Oliveira LS, Rodrigues Fde M, de Oliveira FS, Mesquita PR, Leal DC, et al. (2008) Headspace solid phase microextraction/gas chromatography-mass spectrometry combined to chemometric analysis for volatile organic compounds determination in canine hair: a new tool to detect dog contamination by visceral leishmaniasis. J Chromatogr B Analyt Technol Biomed Life Sci 875: 392-398.

19. Zhang JX, Sun L, Zhang JH, Feng ZY (2008) Sex- and gonad-affecting scent compounds and 3 male pheromones in the rat. Chem Senses 33: 611-621.

20. Gallagher M, Wysocki CJ, Leyden JJ, Spielman AI, Sun X, et al. (2008) Analyses of volatile organic compounds from human skin. Br J Dermatol 159: 780-791.

21. Syed Z, Leal WS (2009) Acute olfactory response of *Culex* mosquitoes to a human- and bird-derived attractant. Proc Natl Acad Sci USA 106: 18803-18808.

22. Degreff LE, Curran AM, Furton KG (2011) Evaluation of selected sorbent materials for the collection of volatile organic compounds related to human scent using non-contact sampling mode. Forensic Sci Int 209: 133-142.

23. Ara K, Hama M, Akiba S, Koike K, Okisaka K, et al. (2006) Foot odor due to microbial metabolism and its control. Can J Microbiol 52: 357-364.

24. Caroprese A, Gabbanini S, Beltramini C, Lucchi E, Valgimigli L (2009) HS-SPME-GC-MS analysis of body odor to test the efficacy of foot deodorant formulations. Skin Res Technol 15: 503-510.

25. Phillips M, Herrera J, Krishnan S, Zain M, Greenberg J, et al. (1999) Variation in volatile organic compounds in the breath of normal humans. J Chromatogr B Biomed Sci Appl 729: 75-88.

26. Cork A, Park KC (1996) Identification of electrophysiologically-active compounds for the malaria mosquito, *Anopheles gambiae*, in human sweat extracts. Med Vet Entomol 10: 269-276.

27. Zeng X-N, Leyden JJ, Lawley H, Sawano K, Nohara I, et al. (1991) Analysis of characteristic odors from human male axillae. J Chem Ecol 17: 1469-1492.

28. Kusano M, Mendez E, Furton KG (2013) Comparison of the volatile organic compounds from different biological specimens for profiling potential. J Forensic Sci 58: 29-39.

29. Mansson HL (2008) Fatty acids in bovine milk fat. Food Nutr Res 52: 10.

30. Wheatley VR, James AT (1957) Studies of sebum. 7. The composition of the sebum of some common rodents. Biochem J 65: 36-42.

31. Braks MAH, Meijerink J, Takken W (2001) The response of the malaria mosquito, *Anopheles gambiae,* to two components of human sweat, ammonia and L-lactic acid, in an olfactometer. Physiol Entomol 26: 142-148.

32. Dekker T, Steib B, Carde RT, Geier M (2002) L-lactic acid: a human-signifying host cue for the anthropophilic mosquito *Anopheles gambiae*. Med Vet Entomol 16: 91-98.

33. Costantini C, Birkett MA, Gibson G, Ziesmann J, Sagnon NF, et al. (2001) Electroantennogram and behavioural responses of the malaria vector *Anopheles gambiae* to human-specific sweat components. Med Vet Entomol 15: 259-266.

34. Hudson-Holness DT, Furton KG (2010) Comparison between human scent compounds collected on cotton and cotton blend materials for SPME-GC/MS analysis. J Forensic Res 1: 101.

35. Moore JG, Jessop LD, Osborne DN (1987) Gas-chromatographic and mass-spectrometric analysis of the odor of human feces. Gastroenterology 93: 1321-1329.

36. Verhulst NO, Andriessen R, Groenhagen U, Bukovinszkine Kiss G, Schulz S, et al. (2010) Differential attraction of malaria mosquitoes to volatile blends produced by human skin bacteria. PLoS ONE 5: e15829.

37. Kim SK, Karadeniz F (2012) Biological importance and applications of squalene and squalane. Adv Food Nutr Res 65: 223-233.
