# Supplementary material for: Diverse Host-Seeking Behaviors of Skin-Penetrating Nematodes
Source: PLoS Pathog. 2014 Aug 14;10(8):e1004305. doi: 10.1371/journal.ppat.1004305 (PMC4133384; doi:10.1371/journal.ppat.1004305)
Supplement: Table S6 — Comparison of 1 hour and 3 hour chemotaxis assays with Str. ratti . Results from 1 hour and 3 hour assays were not significantly different (two-way ANOVA). (DOCX) [file ppat.1004305.s012.docx]

**Table S6. Comparison of 1 hour and 3 hour chemotaxis assays with *Str. ratti.*** Results from 1 hour and 3 hour assays were not significantly different (two-way ANOVA).

| **Odorant** | **C.I. in 3 hr assay** | | | **C.I. in 1 hr assay** | | |
| --- | --- | --- | --- | --- | --- | --- |
|  | mean | n | SEM | mean | n | SEM |
| 1-nonanol | 0.53 | 10 | 0.10 | 0.48 | 6 | 0.06 |
| 1-octanol | 0.41 | 8 | 0.12 | 0.38 | 6 | 0.12 |
| 7-octenoic acid | 0.40 | 6 | 0.12 | 0.32 | 6 | 0.13 |
| decyl acetate | 0.64 | 10 | 0.08 | 0.47 | 7 | 0.13 |
